# *Plesiomonas shigelloides* Infection in Southeast China

**DOI:** 10.1371/journal.pone.0077877

**Published:** 2013-11-04

**Authors:** Xiao Chen, Yu Chen, Qing Yang, Haishen Kong, Fei Yu, Dongsheng Han, Shufa Zheng, Dawei Cui, Lanjuan Li

**Affiliations:** 1 State Key Laboratory for Diagnosis and Treatment of Infectious Diseases, The First Affiliated Hospital, College of Medicine, Zhejiang University, Hangzhou, Zhejiang, China; 2 Center of Clinical Laboratory, The First Affiliated Hospital, College of Medicine, Zhejiang University, Hangzhou, Zhejiang, China; Zhejiang University, China.

## Abstract

**Background:**

*Plesiomonas shigelloides* can cause gastroenteritis and extra-intestinal diseases in humans. However, the prevalence of *P. shigelloides* infections has not been investigated in China.

**Methods:**

Consecutive fecal specimens from outpatients with acute diarrhea and non-diarrheal patients at nine sentinel hospitals in southeast China were collected from March 2010 to May 2012. Bacterial pathogens were detected by culture, and *P. shigelloides* isolates were subjected to antimicrobial susceptibility testing. We also retrospectively reviewed the hospital microbiology laboratory and infection-control databases for all *P. shigelloides* isolates identified from 2001–2012 at our institution in addition to data on the patients' clinical and demographic characteristics.

**Results:**

A total of 3,536 outpatients with acute diarrhea were enrolled in the study. *P. shigelloides* was isolated from 104 (2.9%) patients and accounted for 7.3% of bacterial isolates. Single-pathogen infections with *P. shigelloides* were present in 76 (73.1%) patients. No strain of *P. shigelloides* was isolated from the 478 non-diarrheal patients. Based on 444,684 nonfecal specimens, eight patients developed *P. shigelloides*-related extra-intestinal infections over the 12-year period. All eight patients had underlying diseases, including four with biliary tract diseases and three with liver diseases. Six cases were classified as nosocomial, and five cases were polymicrobial. *P. shigelloides* was sensitive to most antimicrobial drugs, except ampicillin.

**Conclusions:**

In southeast China, *P. shigelloides* has significant clinical relevance, although the isolation rate is low.

## Introduction

*Plesiomonas shigelloides* is a facultative, anaerobic, gram-negative rod that was recently classified in the family Enterobacteriaceae and is the only oxidase-positive member of this family. Similar to many Enterobacteriaceae species, *P. shigelloides* is found in a wide range of hosts, including cats, cows, dogs, pigs, and monkeys [1]. In humans, *P. shigelloides* has been implicated in gastrointestinal infections [2]–[3], and the organism has also been shown to cause bacteremia, sepsis, meningitis, pneumonia, osteomyelitis, keratitis, and other non-diarrheal diseases [4]–[7]. In a recent extensive review of illnesses caused by *P. shigelloides*, little information was presented about the prevalence of *P. shigelloides* infections in China. We recently observed an association between *P. shigelloides* and infections involving gastroenteritis and extra-intestinal illnesses. Here, we provide a local perspective on the diseases caused by *P. shigelloides* in southeast China.

## Materials and Methods

### Ethics statement

Our study was approved by the Ethical Review Board of the First Affiliated Hospital, College of Medicine, Zhejiang University. Before sampling, we informed the patients (or parents of the patients) about the purpose and significance of the study. If the patients (or parents of the patients) then provided consent, they signed their names at the end of the questionnaire.

### Intestinal infections in outpatients and non-diarrheal patients

Nine hospitals located in different areas of southeast China, including seven general hospitals, one children's hospital, and one community hospital, were selected as surveillance sites. The subjects were outpatients with acute diarrheal disease (defined as three or more watery or loose stools in a 24-h period and lasting less than 14 days) and non-diarrheal patients. The non-diarrheal group of apparently healthy individuals with a similar age distribution was selected from the clinical population of outpatients. These patients were admitted to the hospital for a health examination and had no history of diarrhea and/or use of any antimicrobial agent during the previous 6 days. The patients with acute diarrhea were randomly enrolled each week throughout the year, with no fewer than 50 cases per month. The sentinel hospitals administered the patient questionnaires, collected and packaged the fecal specimens, isolated and identified the bacteria, and stored the isolates. The specimens were frozen at −20°C, and the isolates were stored at −80°C in trypticase soy broth (TSB) containing 20% glycerol. The preserved specimens and isolates were delivered to our laboratory on dry ice every 2 weeks. The identification of diarrheagenic *Escherichia coli* (DEC) and the detection of enteric viruses were performed in our laboratory. Questionnaires covered the demographic characteristics of the participants, their illness symptoms, the results of routine stool tests, and medications taken before their hospital visits. Before the study commenced, the relevant program staff at each sentinel hospital participated in a unified training program. Additionally, the laboratory of every sentinel hospital participated in a quality-control evaluation every 6 months, with the detection of quality-control strains.

Stool specimens were cultured on selective media and in enrichment broths to detect Salmonella spp., Shigella spp., Aeromonas spp., Campylobacter spp., Vibrio cholerae, Vibrio parahaemolyticus, P. shigelloides, Yersinia enterocolitica, and suggestive E. coli immediately upon arrival at the laboratories of the sentinel hospitals. All the isolates except suggestive E. coli were confirmed with the VITEK 2 Compact bacterial identification system (bioMerieux, Marcy l'Etoile, France) or API strips (bioMerieux, Marcy l'Etoile, France). The processes for selecting suggestive E. coli and DEC strain screening were in accordance with previous methods described by our study group [8].

### Intestinal infections in inpatients and extra-intestinal infections

Our institution is a 2,500-bed teaching and tertiary care facility in southeast China. The hospital microbiology laboratory and infection-control databases were reviewed for all *P. shigelloides* isolates identified from 2001 through 2012. Data from patients from whom isolates were recovered were investigated for evidence of infection based on the following criteria: (1) fever, (2) radiographic evidence, (3) altered white blood cell counts, and (4) positive *P. shigelloides* cultures. Data regarding the patients' clinical and demographic characteristics were obtained retrospectively by a review of the patients' medical records. Infections were identified as either community-acquired or nosocomial. The latter infections were defined as having occurred >24 h after admission, with signs and symptoms of infection that were absent at the time of admission [9]. Nosocomial postoperative infections were those infections acquired within 30 days of a surgical procedure [9], [10].

### Antimicrobial susceptibility testing

Antimicrobial susceptibility testing was performed on 97 *P. shigelloides* strains using the Kirby-Bauer disc-diffusion method. *E. coli* ATCC 25922 and *Pseudomonas aeruginosa* ATCC 27853 were used as controls in the antimicrobial susceptibility tests. The results were analyzed with WHONET 5.6 software, referring to the breakpoints of CLSI 2011 [11].

### Statistical analyses

Statistical analyses were performed using the Statistical Package for Social Sciences version 17.0 (SPSS, Inc., Chicago, IL, USA). The statistical significance of the differences between groups was determined with the χ^2^-test or Fisher's exact test. All reported *P*-values were two-sided, and *P*<0.05 was considered statistically significant.

## Results

A total of 3,536 outpatients with acute diarrhea from March 2010 to May 2012 were enrolled in the study. There were 1,964 males and 1,572 females, ranging in age from 1 month to 94 years. *P. shigelloides* was isolated from 104 (104/3,536, 2.9%) patients with acute diarrhea and accounted for 7.3% of bacterial isolates. The *P. shigelloides* isolation rate ranked fourth among bacterial pathogens; in descending order, the top three were DEC, *V. parahaemolyticus*, and *Aeromonas spp.* (Table 1). Patients with *P. shigelloides* infections ranged in age from 3 months to 86 years; more than half were 19–44 years old, and nearly one third were 45–59 years old. There was no significant difference between males and females with respect to the incidence of *P. shigelloides*. *P. shigelloides* was isolated throughout the year; however, the peak season for the disease was June to September (Table 2). Sixteen (16/104, 15.4%) patients had a history of consuming seafood or uncooked food in the week preceding their illness. Five (5/104, 4.8%) patients reported that relatives or friends who shared the same meal also had diarrhea. Of the 104 patients infected with *P. shigelloides*, single infections were reported in 76 patients (73.1%), and co-infections with other pathogens were reported in 28 patients (26.9%) (Table 1). The other pathogens isolated included *V. parahaemolyticus* (11 isolates), *Aeromonas spp.* (8 isolates), enteroaggregative *E. coli* (4 isolates), enterotoxigenic *E. coli* (2 isolates), enteropathogenic *E. coli* (1 isolate), *Salmonella enteritidis* (1 isolate), and *Y. enterocolitica* (1 isolate). The symptoms of patients with single *P. shigelloides* infections differed from the symptoms of co-infected patients; however, the differences were not significant (Table 3).

**Table 1:** Prevalence of bacterial pathogens in patients with acute diarrhea and those without diarrhea during the period of March 2010 to May 2012.

Bacterial isolate	No. (%) of cases		*P*
	Diarrhea (n = 3,536)	Non-diarrheal (n = 478)	
Single infection with *Plesiomonas shigelloides*	76 (2.1)	0	0.001
*Plesiomonas shigelloides* and co-infection with any other pathogen	28 (0.8)	0	0.097
Total *Plesiomonas shigelloides* infections	104 (2.9)	0	0.000
Diarrheagenic *Escherichia coli*	594 (16.8)	55 (11.5)	0.003
Enteroaggregative *E. coli*	363 (10.3)	30 (6.3)	0.006
Enterotoxigenic *E. coli*	115 (3.3)	12 (2.5)	0.384
Enteropathogenic *E. coli*	97 (2.7)	12 (2.5)	0.769
Shiga toxin-producing *E. coli*	15 (0.4)	0	0.304
Enteroinvasive *E. coli*	4 (0.1)	1 (0.2)	0.470
*Vibrio parahaemolyticus*	315 (8.9)	3 (0.6)	0.000
*Aeromonas spp.*	152 (4.3)	5 (1.1)	0.001
*Shigella spp.*	57 (1.6)	0	0.005
*Salmonella spp.*	39 (1.1)	0	0.040
*Vibrio cholerae* (non O1/O139 serogroup)	25 (0.7)	1 (0.2)	0.332
*Yersinia enterocolitica*	21 (0.6)	1 (0.2)	0.460
*Campylobacter spp.*	17 (0.5)	1 (0.2)	0.639

**Table 2:** Demographic characteristics of patients with *Plesiomonas shigelloides* infection and acute diarrhea.

Variable	Category	No. of samples tested	No. (%) of positive samples	*P*
Time				<0.001
	March to May	591	9 (1.5)	
	June to August	1564	69 (4.4)	
	September to November	823	24 (2.9)	
	December to February	558	2 (0.4)	
Age (years)				<0.001
	0–5	930	8 (0.9)	
	6–18	205	4 (1.9)	
	19–44	1137	54 (4.7)	
	45–59	837	29 (3.5)	
	≥60	427	9 (2.1)	
Sex				0.517
	Male	1964	61 (3.1)	
	Female	1572	43 (2.7)	

**Table 3:** Symptoms of *Plesiomonas shigelloides* infection with acute diarrhea.

Symptoms	No. (%) of cases		*P*
	Singly infected (n = 76)	Co-infected (n = 28)	
Watery diarrhea	53 (69.7)	17 (60.7)	0.384
Bloody diarrhea	11 (14.5)	6 (21.4)	0.581
Visible mucus in stool	13 (17.1)	7 (25.0)	0.365
Fever	22 (28.9)	12 (42.9)	0.180
Vomiting	9 (11.8)	5 (17.9)	0.636
Abdominal pain	50 (65.8)	20 (71.4)	0.587

A total of 478 non-diarrheal patients were enrolled in the study from March 2010 to May 2012. There were 282 males and 196 females, with ages ranging from 5 months to 87 years (95 [19.9%] were ≤5 years old, 39 [8.2%] were 6–18 years old, 179 [37.4%] were 19–44 years old, 121 [25.3%] were 45–59 years old, and 44 [9.2%] were >60 years old). *P. shigelloides* strains were not isolated from the non-diarrheal patients. The *P. shigelloides* isolation rate was significantly different from that in patients with acute diarrhea (*P* = 0.000) (Table 1).

A total of 22,105 stools obtained from inpatients at our institution from 2001–2012 were analyzed retrospectively, and only two specimens (2/22,105, 0.009%) were positive for *P. shigelloides*. Both cases occurred in August. These two patients had clinical manifestations of gastroenteritis and underlying diseases: one had viral hepatitis B-related cirrhosis and the other had diabetes mellitus. The patient with liver disease also developed *Aeromonas hydrophila* septicemia.

From 2001–2012, a total of 444,684 nonfecal specimens at our institution were analyzed retrospectively, including 161,818 blood samples, 125,073 sputum samples, 60,184 urine samples, 16,923 abdominal fluid samples, 14,376 drainage fluid samples, 10,701 throat swabs, 7,065 secretions, 6,800 cerebrospinal fluid samples, 5,787 bile samples, 5,610 pleural fluid samples, 2,489 abscesses, 446 organ samples, and 27,412 other samples. During the study period, eight patients (8/444,684, 0.0018%) developed *P. shigelloides-*related extra-intestinal infections (Table 4). Most cases (6/8, 75.0%) occurred during the late spring and summer (May to August), and most patients were over 55 years old (7/8, 87.5%). All eight patients had underlying diseases, including biliary tract diseases, which were present in four patients, and immunosuppressive medical conditions, which were present in four patients (two patients had viral hepatitis B, one had duodenal malignancies, and one had hypertensive renal disease). All eight patients had fever and elevated total white cell counts or neutrophil levels. In six (75%) of the eight patients, the *P. shigelloides* infections were classified as nosocomial, and the infections occurred after surgery in five patients. In five (62.5%) of the eight patients, the *P. shigelloides* infections were polymicrobial, regardless of whether the isolates were obtained from blood or non-blood samples. In two patients, *P. shigelloides* and *E. coli* were isolated simultaneously from the same blood specimen. Three patients developed septicemia as a complication of infection, although all these patients recovered without sequelae. Of the patients who were cured and whose *P. shigelloides* isolates were tested for susceptibility to the antimicrobial agent or agents received, all had received at least one agent active against the *P. shigelloides* strain or another isolated strain.

**Table 4:** Features of eight patients with *Plesiomonas shigelloides* extra-intestinal infections.

No.	Age	Sex	Predisposing factor(s)		Comorbidities	Clinical presentation	Pathogen(s) isolated by specimen cultured^a^		Nosocomia etiology	Drug(s)^b^	Outcome
	years		Disease(s) and/or condition(s)	Procedure(s)			Non-blood	Blood			
1	34	M	None	Chemotherapy	Viral hepatitis B, Non-Hodgkin's lymphoma	Septicemia	ND	*P. shigelloides*, *E. coli*	No	CFP-Sulb	Cured
2	68	F	None	None	Viral hepatitis B-related cirrhosis, ascites	Septicemia	ND	*P. shigelloides*	Yes	LEV	Cured
3	60	M	Cholelithiasis	ERCP performed 2 days before episode	None	Cholangitis, septicemia	Bile: *P. shigelloides*	*E. coli*	Yes	IPM	Cured
4	71	F	Cholangitis	None	Hypertension, cholecystectomy 16 years ago	Biliary peritonitis	ND	*P. shigelloides, E. coli*	No	LEV, CRO, tinidazole	Cured
5	55	M	Cerebral hemorrhage	Intracerebral hematoma cleared 5 days before episode	Hypertension, hypertensive renal disease	Pulmonary infection	Sputum: *P. shigelloides*	None	Yes	IPM, LEV	Cured
6	69	F	Gallbladder carcinoma	Cholecystectomy complicated with bile leakage, followed by intestinal and biliary drainage operation	None	Septicemia, surgical site infection	Drainage fluid: *P. Shigelloides, S. marcescens, S. maltophilia, A. baumannii*	*K. pneumoniae*	Yes	CFP-Sulb, IPM, CIP	Cured
7	59	F	Duodenum malignancies	Whipple procedure	None	Surgical site infection	Drainage fluid: *P. shigelloides*	None	Yes	IPM	Cured
8	75	M	Cholelithiasis	Cholecystectomy	Hepatic malignancies	Pulmonary infection	Sputum: *P. shigelloides, S. marcescens, P. aeruginosa*	ND	Yes	IPM, CFP-Sulb	Cured

ERCP, endoscopic retrograde cholangiopancreatography; ND, not done (i.e., specimens were not obtained for culture).

a P. shigelloides, Plesiomonas shigelloides; E. coli, Escherichia coli; S. marcescens, Serratia marcescens; S. maltophilia, Stenotrophomonas maltophilia; A. baumannii, Acinetobacter baumannii; K. pneumoniae, Klebsiella pneumoniae; P. aeruginosa, Pseudomonas aeruginosa.

b MEM, meropenem; CIP, ciprofloxacin; CFP-Sulb, cefoperazone-sulbactam; LEV, levofloxacin; IPM, imipenem; CRO, ceftriaxone.

Of the 97 *P. shigelloides* isolates, most were resistant to ampicillin (91.7%), followed by amikacin (46.2%), trimethoprim/sulfamethoxazole (41.2%), gentamicin (37.1%), and cefazolin (16.0%). However, less than 8.5% were resistant to piperacillin, amoxicillin/clavulanic acid, ampicillin/sulbactam, cefuroxime, ceftazidime, cefotaxime, cefepime, cefoxitin, aztreonam, ciprofloxacin, levofloxacin, or tetracycline. None of the isolates was resistant to cefoperazone/sulbactam, piperacillin/tazobactam, imipenem, or meropenem (Table 5).

**Table 5:** Antimicrobial susceptibility of *Plesiomonas shigelloides*.

Antimicrobial agent	R%^a^	I%^a^	S%^a^
Ampicillin	91.7	4.2	4.1
Piperacillin	7.8	14.4	77.8
Amoxicillin/Clavulanic acid	3.2	1.1	95.7
Cefoperazone/Sulbactam	0.0	3.3	96.7
Ampicillin/Sulbactam	4.4	2.2	93.4
Piperacillin/Tazobactam	0.0	0.0	100.0
Cefazolin	16.0	48.9	35.1
Cefuroxime	3.3	1.1	95.6
Ceftazidime	3.1	2.1	94.8
Cefotaxime	1.1	2.1	96.8
Cefepime	2.1	0.0	97.9
Cefoxitin	6.4	1.3	92.3
Aztreonam	4.3	8.6	87.1
Imipenem	0.0	0.0	100.0
Meropenem	0.0	0.0	100.0
Amikacin	46.2	22.6	31.2
Gentamicin	37.1	32.0	30.9
Ciprofloxacin	8.5	28.7	62.8
Levofloxacin	3.2	0.0	96.8
Trimethoprim/Sulfamethoxazole	41.2	3.1	55.7
Tetracycline	4.5	8.9	86.6

a R, resistant; I, intermediate; S, susceptible.

## Discussion

*P. shigelloides* is an emerging pathogen that is widespread in the aquatic environment. The natural reservoirs of this organism are water, fish, and seafood in temperate and tropical climates [12]–[13]. Southeast China, with its long coastline, is rich in water resources. In this study, we investigated the prevalence of *P. shigelloides* in this region.

The rate of isolation of *P. shigelloides* from the fecal specimens of outpatients with acute diarrhea in this study was 2.9% (accounting for 7.3% of bacterial isolates), which was significantly lower than the rate of 11.4% reported in rural communities in northwestern Ecuador from 2004–2008 [2] and the rate of 10% reported in a remote rural area in western Thailand from 2001–2002 [3], but higher than the rate of 1.7% reported in southeast Nigeria [14]. These results indicate that the distribution of *P. shigelloides* differs greatly between regions and time periods. In our study, the prevalence of *P. shigelloides* in inpatients with diarrhea during the period of 2001–2012 was 0.009%, which was significantly lower than the rate of 2.9% in outpatients during the period of 2010–2012. Apart from the difference in the sample sources, changes in the detection methods might also underlie this discrepancy. Because enteric pathogens were neglected during the period of 2001–2009, stool specimens from inpatients were only cultured on blood agar plates and Salmonella-Shigella agar, and pathogen isolation focused mainly on *Salmonella* and *Shigella*. However, from 2010–2012, stool specimens were cultured on selective media and in enrichment broths to detect more enteric pathogens. In our study, no *P. shigelloides* strains were isolated from the non-diarrheal patients. In a Japanese study, *P. shigelloides* was isolated from <0.1% of asymptomatic individuals [15]. However, a carrier rate as high as 4.2% has been reported in northwestern Ecuador [2], and a rate of 11% was reported in western Thailand [3]. The most likely explanation for this result is the higher prevalence of *P. shigelloides-*associated diarrhea in these regions. These results imply that *P. shigelloides* is not an indigenous organism of the human gastrointestinal tract. *P. shigelloides* has also occasionally caused extra-intestinal diseases. In our study, only eight (0.0018%) patients developed *P. shigelloides*-related extra-intestinal infections over the 12-year period. However, data on the prevalence of extra-intestinal infections caused by *P. shigelloides* are limited and focus primarily on case reports [4]–[7]. At a Hong Kong hospital, a total of seven patients developed *P. shigelloides* bacteremia during a 9-year period [16]. In our study, the *P. shigelloides* isolation rate in patients with acute diarrhea was much higher than in asymptomatic individuals, implying that *P. shigelloides* may be a diarrhea-causing pathogen in patients in southeast China. However, in China, pathogen detection in patients with diarrhea only involves tests for *Salmonella spp., Shigella spp.*, and the *V. cholerae* O1 and O139 groups at present. Our study found that isolates of *Salmonella spp., Shigella spp.*, and *V. cholerae* O1 and O139 groups accounted for only 6.9% of bacterial pathogens. Therefore, it is necessary to test for more pathogens, including *P. shigelloides*, in patients with diarrhea in future routine clinical practice.

In this study, an increase in the number of *P. shigelloides* infections was observed during the summer months. This seasonal variation has also been observed in Hong Kong [17] and Bangladesh [18], and there are also seasonal variations in the numbers of organisms detectable in surface water samples [18]. However, in our study, 15.4% of patients had a history of consuming seafood or uncooked food in the week preceding their illness, and 4.8% of patients reported that their relatives or friends who shared the same meal also had diarrhea. Holmberg *et al.* reported that foreign travel and the consumption of raw shellfish (usually oysters) were two factors strongly associated with *P. shigelloides* infection [19]. There have also been reports of outbreaks attributed to the consumption of freshwater fish in Zaire [20] and contaminated raw oysters and shellfish in the United States [21]. These facts imply that the most common sources of *P. shigelloides* intestinal infections may be contaminated water and raw seafood.

In a case-control study in Ecuador, Escobar *et al.* reported that there was little evidence that single infections with *P. shigelloides* were associated with diarrhea and stronger evidence that co-infections with other pathogens caused diarrhea [2]. However, in our study, co-infections with *P. shigelloides* and other pathogens accounted for only 26.9% of cases. This co-infection analysis might be limited by the number of pathogens considered. Given the data from the literature, it might be useful to consider other pathogens, including *Giardia sp.*, *Entaemoeba histolytica*, rotavirus, and *Cryptosporidium spp.* [2]. The pathogenic capacity of *P. shigelloides* to cause diarrhea is unclear, and more data are required to verify the complex relationships involved.

The isolation of co-pathogens in patients with *P. shigelloides* extra-intestinal infections was relatively common in our study. This phenomenon has also been reported by other researchers [6], [16]. Of course, it is difficult to assess the contribution of each organism to the disease state of patients with polymicrobial infections. In our study, a member of the family Enterobacteriaceae (usually *E. coli*) was always isolated in association with *P. shigelloides*. Woo *et al.* reported that *Enterococcus faecalis* and *P. shigelloides* were isolated simultaneously from dialysis effluent [6]. Patients with *P. shigelloides* extra-intestinal and intestinal infections also showed the same seasonal variability in our study. These results suggest that *P. shigelloides* infections may have an intestinal source.

*P. shigelloides* occasionally causes extra-intestinal diseases, and Woo *et al.* reported that *P. shigelloides* infections were associated with underlying biliary tract diseases [16]. In our study, biliary tract diseases were present in four patients, three of whom suffered from septicemia as a complication of the infection. Bacteria can enter the biliary tract by ascending the gastrointestinal tract, although hematogenous spread via the portal venous blood may also play a role [22]. Sphincterotomy and biliary stenting are recognized risk factors for contamination of the biliary tract with bacteria [22], and *P. shigelloides* may gain access to the biliary tree via this route if the organism is present in the gastrointestinal tract. One patient (patient 3) in our study had undergone endoscopic retrograde cholangiopancreatography (ERCP) 2 days before the onset of infection. Apart from underlying biliary tract diseases, three patients in our study had liver disease: one (patient 1) had viral hepatitis B, one (patient 2) had viral hepatitis B-related cirrhosis, and one (patient 8) had hepatic malignancies. Thus, *P. shigelloides* might be associated with liver disease. Because there is no apparent change in the lower-intestinal bacterial flora of patients with cirrhosis [23], *P. shigelloides* may be particularly virulent in these patients, and such species account for a larger percentage of the total bacterial population in individuals with gram-negative bacteremia and cirrhosis compared with those without cirrhosis [24]. In contrast, six of eight patients were classified as having nosocomial infections in our study. Unlike community-acquired infections, the sources of nosocomial infections are often unknown.

The antibiotic susceptibility patterns of our isolates were typical of the patterns reported by others [17], [25]. However, *P. shigelloides* appeared to have reduced susceptibility to amikacin and gentamicin. Antibiotic therapy is not usually necessary for the management of intestinal infections with *P shigelloides* because the illness is self-limiting in most cases [26]. Nevertheless, the infection responds well to antibiotic therapy, and such therapies reduce the duration of the illness compared with its duration in untreated patients [27]. Therefore, diarrhea patients with severe and protracted symptoms should receive antibiotic therapy. Patients with extra-intestinal infections may also benefit from antibiotic therapy. All patients with extra-intestinal infections in this study were prescribed antibiotics empirically and recovered without any sequelae. It is important for doctors to understand the characteristic antibiotic susceptibility of *P. shigelloides* if they are to select the appropriate antimicrobial agent for empirical treatments.

In conclusion, *P. shigelloides* is not an indigenous organism of the human gastrointestinal tract. In southeast China, *P. shigelloides* might be a pathogen in diarrhea patients that is contracted by ingesting contaminated water or raw seafood, and it is necessary to routinely test for this pathogen in future clinical work. *P. shigelloides* occasionally causes extra-intestinal infections, which are associated with underlying biliary tract and liver diseases.
